# Pinning of a ferroelectric Bloch wall at a paraelectric layer

**DOI:** 10.3762/bjnano.9.220

**Published:** 2018-08-31

**Authors:** Vilgelmina Stepkova, Jiří Hlinka

**Affiliations:** 1 Institute of Physics, The Czech Academy of Sciences, 182 21 Prague, Czech Republic

**Keywords:** BaTiO_3_–SrTiO_3_ superlattices, ferroelectric domain walls, Ginzburg–Landau–Devonshire model, phase-field simulations

## Abstract

The phase-field simulations of ferroelectric Bloch domain walls in BaTiO_3_–SrTiO_3_ crystalline superlattices performed in this study suggest that a paraelectric layer with a thickness comparable to the thickness of the domain wall itself can act as an efficient pinning layer. At the same time, such a layer facilitates the possibility to switch domain wall helicity by an external electric field or even to completely change the characteristic structure of a ferroelectric Bloch wall passing through it. Thus, ferroelectric Bloch domain walls are shown to be ideal nanoscale objects with switchable properties. The reported results hint towards the possibility to exploit ferroelectric domain wall interaction with simple nanoscale devices.

## Introduction

Nanometer-scale mixtures of paraelectric and ferroelectric materials in disordered solid solutions or in very fine artificial crystalline superlattices often show qualitatively similar domain phenomena as the parent ferroelectric materials. For example, in the case of BaTiO_3_–SrTiO_3_ superlattices with only a few atomic layers of SrTiO_3_, the domain walls are simply expected to penetrate through BaTiO_3_/SrTiO_3_ interfaces [1–5]. In general, it can be expected that a small amount of paraelectric defects, smaller or thinner than the correlation length, will not substantially alter the superposed domain structure in a strong ferroelectric material like BaTiO_3_. In other words, a sufficiently thin paraelectric layer is effectively polarized by the neighboring ferroelectric material. However, little is known about robustness of the inner polarization, present within the nanoscale thickness of ferroelectric Bloch walls [6–7]. For example, it has not yet been clarified whether such a localized polarization is sustained under the influence of the chemical stoichiometry concentration fluctuations typical for relaxor ferroelectric perovskites, for example. Similarly, we are not aware of any device geometries that can define or alter the helicity of Bloch walls.

**Figure 1:**
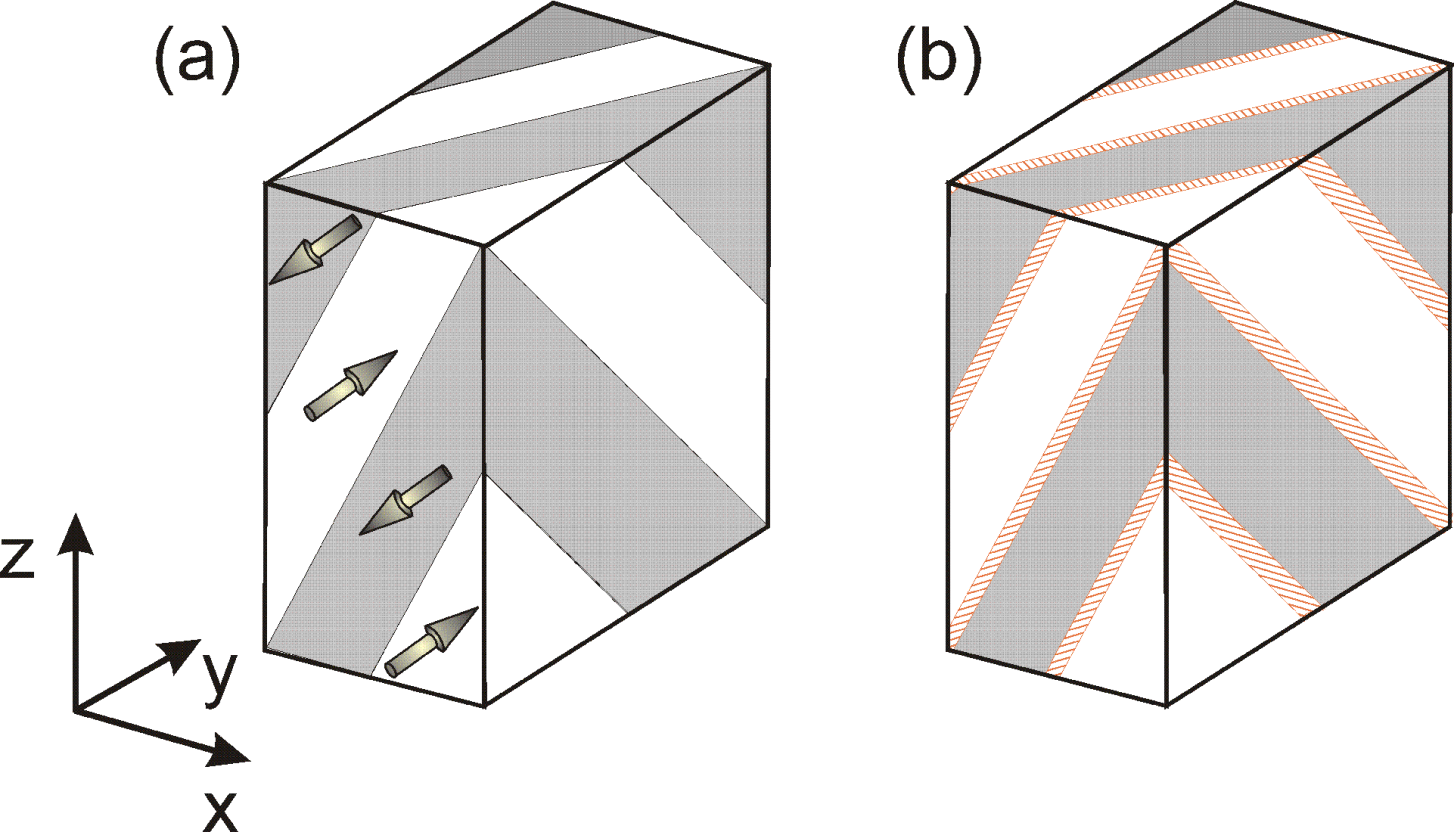
Domain structure with 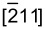-oriented 180-degree domain walls in a) pure BaTiO_3_, b) BaTiO_3_–SrTiO_3_ superlattice with SrTiO_3_ layers situated within the domain walls. Shaded areas correspond to [111] polarized domains, unshaded areas represent 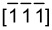 domains. Hatched regions in b) correspond to SrTiO_3_ layers.

In order to assess the interaction of Bloch walls with material inhomogeneities, we have explored the limiting case of ferroelectric Bloch walls encountering a layer of paraelectric material by means of phase-field simulation. This technique allows the relaxed domain wall profiles to be predicted by simulated annealing of the system based on numerical solution of material-specific time-dependent Ginzburg–Landau–Devonshire equations [8–10]. It is intuitively clear that a paraelectric layer would act as pinning loci for the Ising ferroelectric wall because the interior of an Ising wall is not polarized at all. In that respect, the largest effect is expected for a layer with thickness matching that of the domain wall. Therefore, we have considered a hypothetical BaTiO_3_–SrTiO_3_ crystalline superlattice, formed by thin SrTiO_3_ paraelectric layers of 0.5–3 nm thickness, separated by about 13 nm thick BaTiO_3_ ferroelectric slabs (see Figure 1). The SrTiO_3_ layers were normal to the 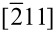 crystallographic direction, common to the parent cubic lattice of both BaTiO_3_ and SrTiO_3_.

**Figure 2:**
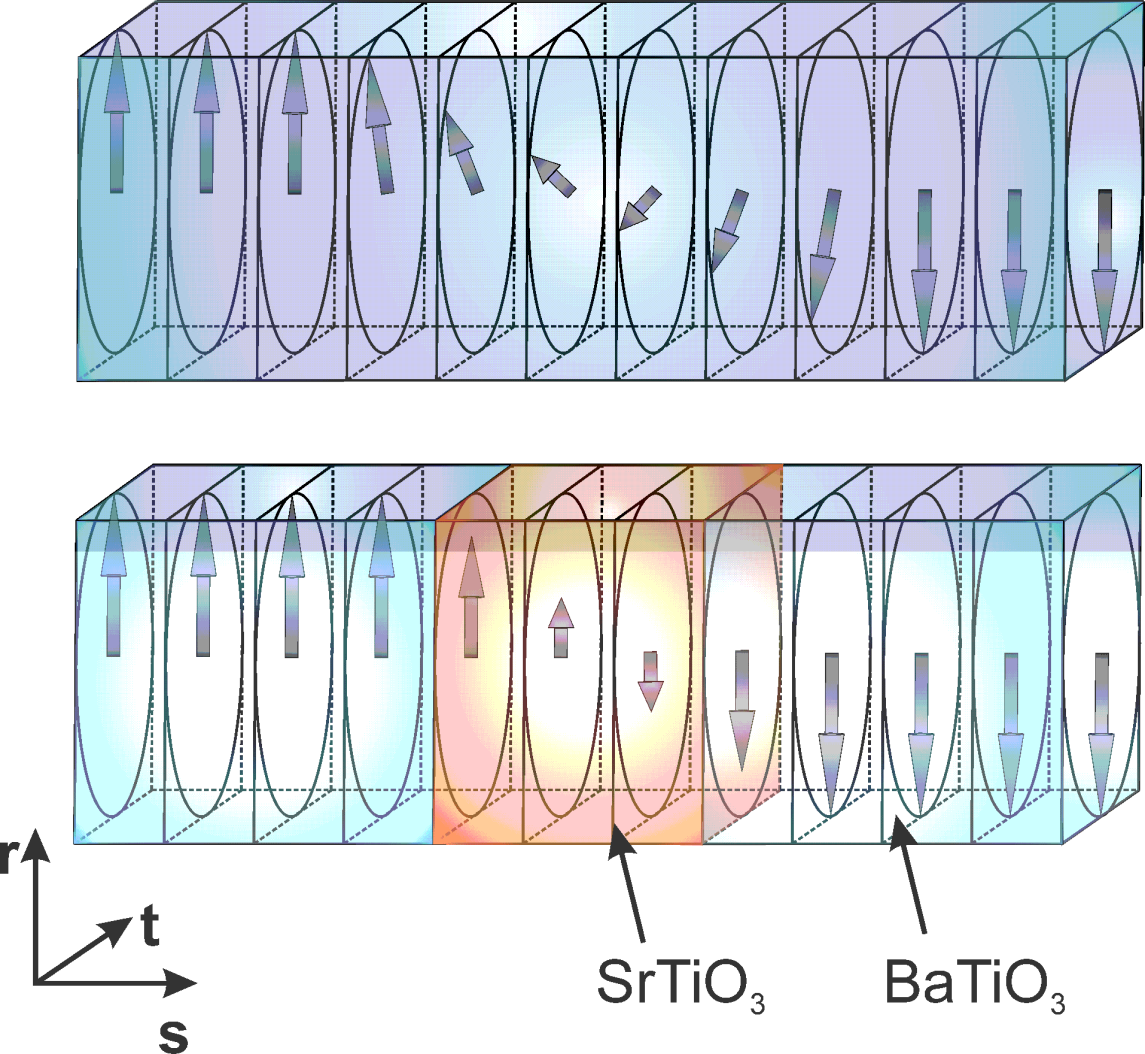
Schematic illustration of polarization passing across the 180-degree domain wall in pure rhombohedral BaTiO_3_ and in the superlattice containing 1 nm thin SrTiO_3_ layers. Note the almost Ising-like profile in the bottom panel.

It is found that a nanometer thin layer of SrTiO_3_ acts as a pinning loci not only for the Ising wall but also for the Bloch wall. Moreover, these results suggest that such a pinned Bloch wall can loose practically all of its inner polarization (see Figure 2). This result could be possibly used to set or modify the helicity of Bloch walls passing through conveniently placed paraelectric gate layers in future domain-wall-based devices.

## Results and Discussion

The peculiarity of the investigated ferroelectric domain wall is best understood when the polarization is expressed in the symmetry-adapted Cartesian system [6] associated with the set of orthogonal unit vectors **r** || [111], **s** || 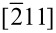, and **t** || 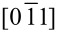. By definition [6], the adjacent domains differ in the sign of the *P*_r_ component. In the case of Ising walls, the integral of the *P*_t_ component across a given wall is zero. In the case of Bloch walls, there is an overall polarization in the *P*_t_ component within the few nanometer thickness of the given domain wall itself. This inner polarization can be negative or positive.

**Figure 3:**
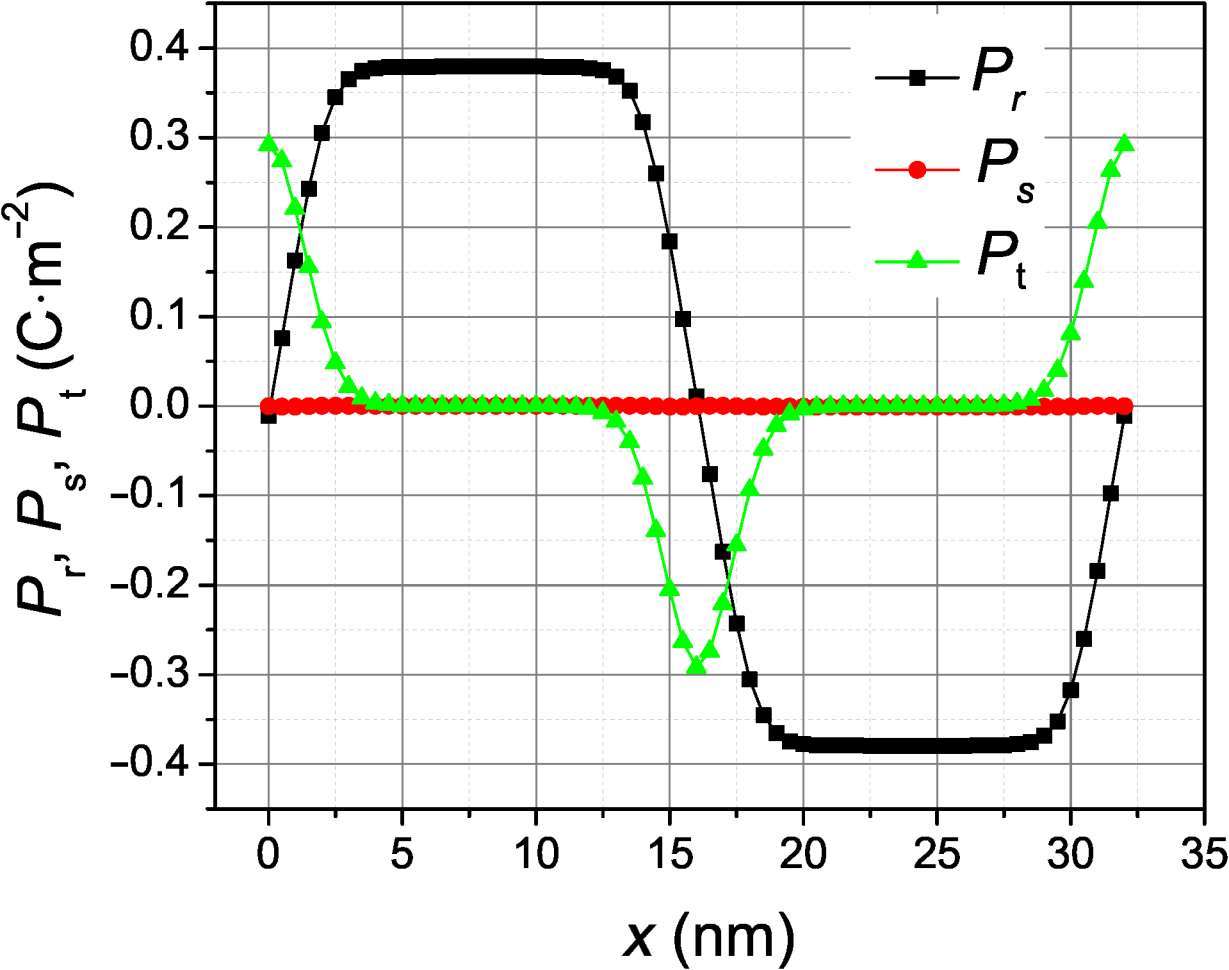
Relaxed profile of the polarization components across 180-degree ferroelectric wall in rhombohedral BaTiO_3_.

**Figure 4:**
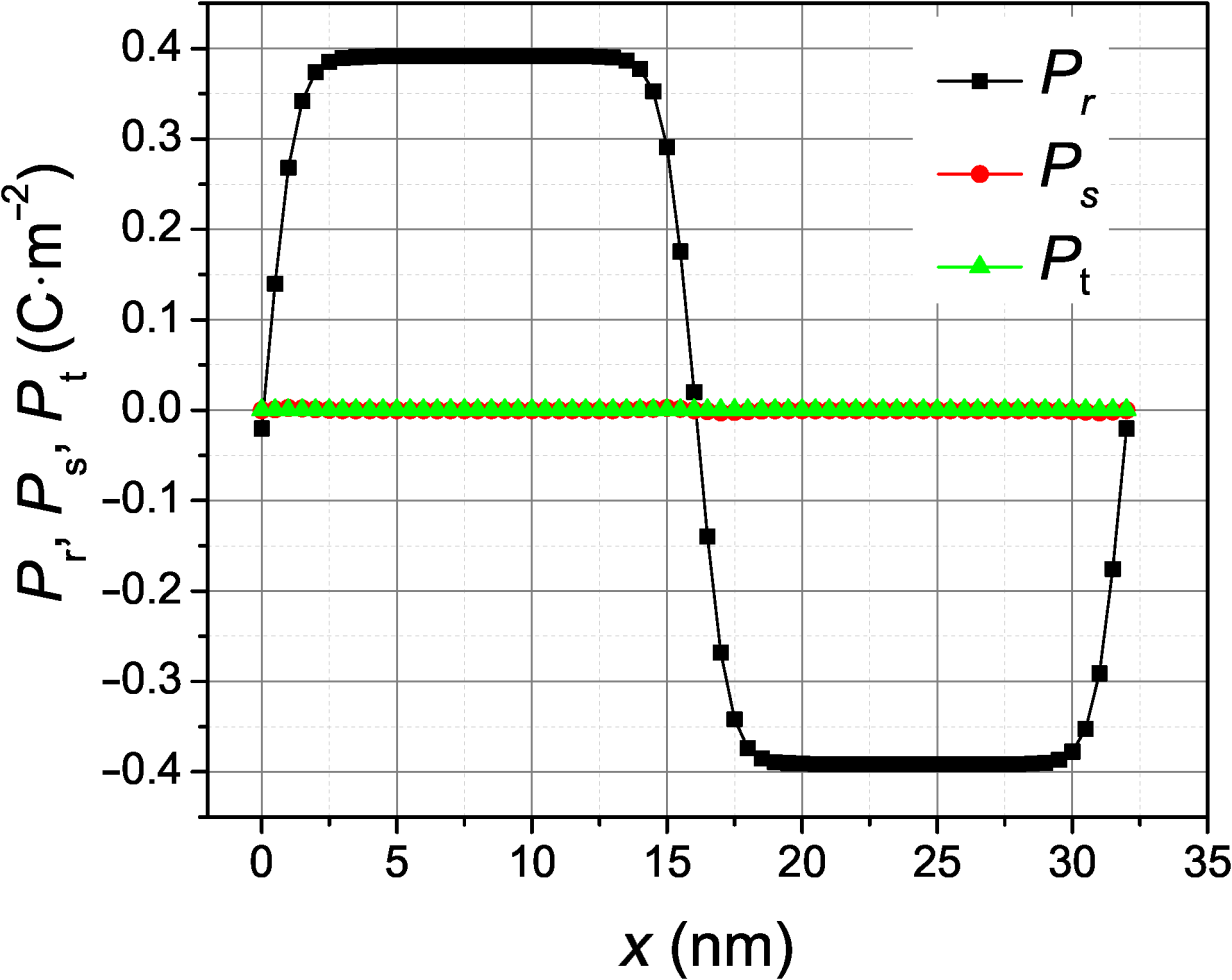
Relaxed profile of the polarization components across 180-degree ferroelectric wall in rhombohedral BaTiO_3_ under epitaxial compressive stress of 3 GPa.

The relaxed, equilibrium polarization profile in the simulation for pure BaTiO_3_ is shown in Figure 3. The nonzero *P*_t_ peak located at the wall implies that these domain boundaries are indeed Bloch walls. The alternating sign of the *P*_t_ component in subsequent domain walls indicates that the energetically equivalent Bloch walls present in this simulation have the same helicity. For comparison, Figure 4 shows the profile of the Ising domain wall, which is obtained by the same simulation but under an epitaxial compressive stress of 3.0 GPa, applied in the plane perpendicular to the spontaneous polarization as described in [11].

**Figure 5:**
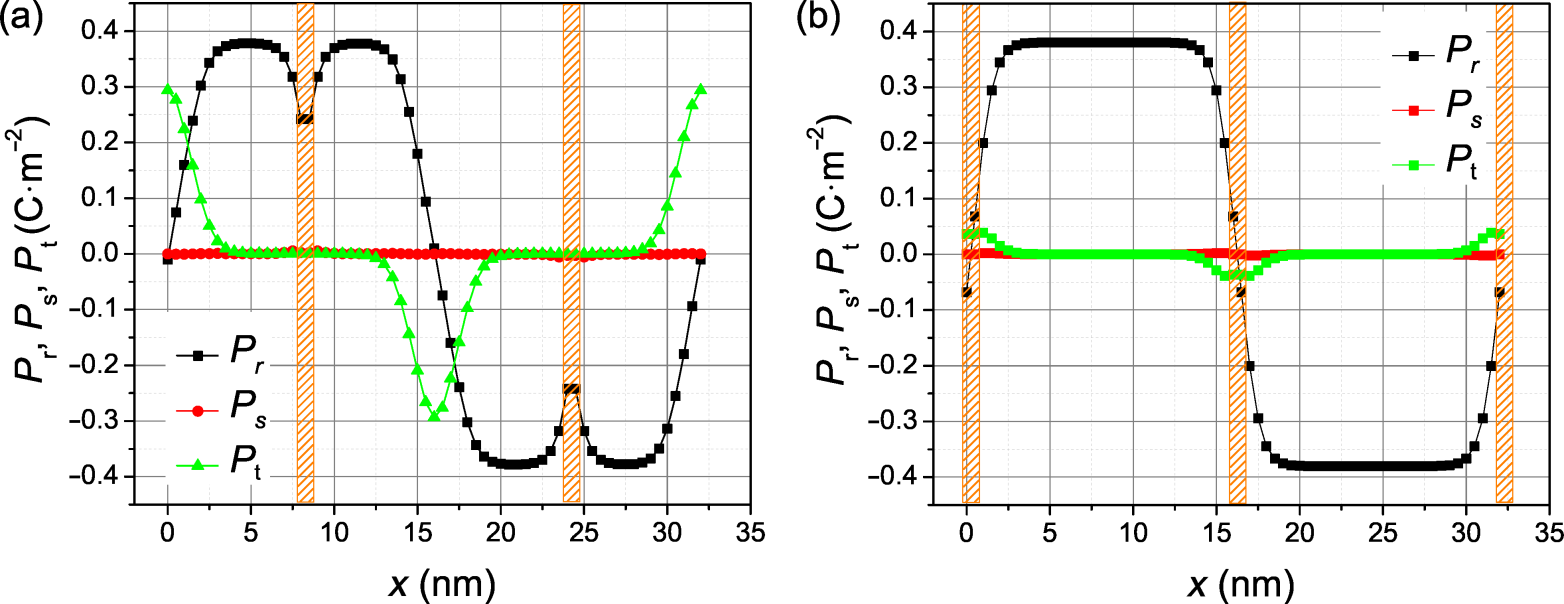
Relaxed profile of the polarization components across 180-degree ferroelectric walls in rhombohedral BaTiO_3_ intercalated with 1 nm thin layers of SrTiO_3_. (a) SrTiO_3_ layer within the ferroelectric domain. (b) SrTiO_3_ layer located at the ferroelectric domain wall.

The polarization profile relaxed within the SrTiO_3_-containing superlattice layers as shown in Figure 5. When the domain wall is far away from the SrTiO_3_ layer, the domain wall profile is barely modified, only the spontaneous polarization in the domain is somewhat reduced (by about 40%, see Figure 5a). In contrast, when the domain wall happens to be located right at the SrTiO_3_ layer, the *P*_t_ component is suppressed considerably there (by more than 80% in Figure 5b). In other words, its Bloch character is strongly suppressed.

**Figure 6:**
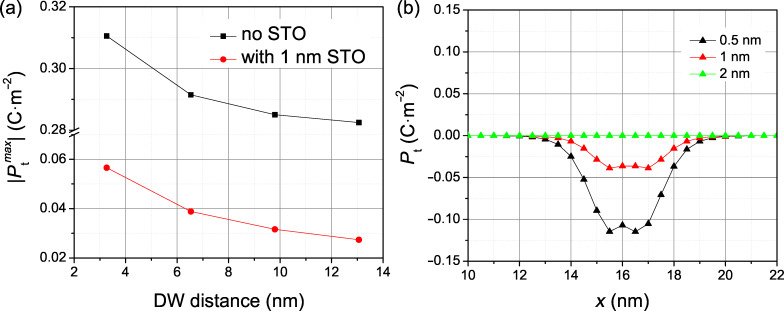
Variation of the *P*_t_ component (a) with increasing distance between domain walls and (b) with the thickness of the SrTiO_3_ layer.

Similar calculations made in supercells of different sizes (Figure 6a) show a rather marginal dependence of the domain wall shape on the distance between domain walls. As a matter of fact, the suppression of *P*_t_ is a marked effect even when the next domain wall is only 4 nm away. The monotonous dependence shown in Figure 6a demonstrates that, for larger distances between domain walls, the amplitude ratio between the *P*_t_ component of the wall located at the SrTiO_3_ layer and far away from it is even stronger. On the other hand, as expected, the suppression of the *P*_t_ component is very sensitive to the thickness of the SrTiO_3_ layer. As is apparent from Figure 6b, as few as 2 nm of SrTiO_3_ is in fact sufficient to suppress *P*_t_ completely.

**Figure 7:**
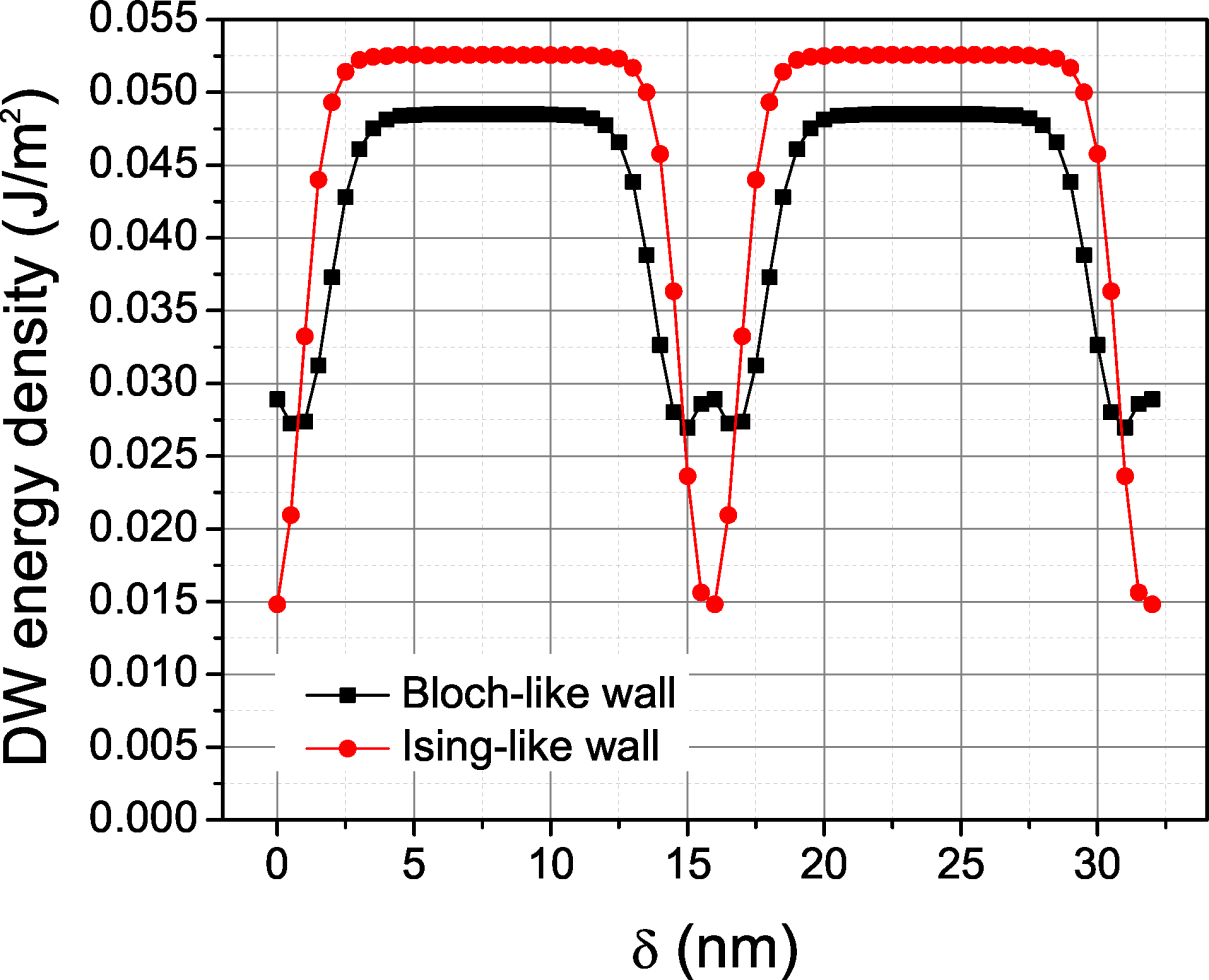
Landau part of the domain wall energy density as a function of the position of the ferroelectric domain wall, estimated by sliding a rigid polarization profile across the potential relief of the superlattice described in the text.

In order to appreciate the height and shape of the well-formed potential by the 1 nm SrTiO_3_ layer, we have also calculated the Landau energy contribution to the domain wall energy density as a function of the position of the domain wall, assuming that the shape of the profile of the domain wall is not modified while sliding across the SrTiO_3_ layer. The result of this calculation, obtained numerically by integrating the Landau energy density, is shown in Figure 7. The calculation was made for the ideal profile of the Ising wall and Bloch wall of Figure 3 and Figure 4, subtracting the single-domain energy density in pure BaTiO_3_ as a reference energy scale. Since the SrTiO_3_ layer is thinner than the domain wall width, the width of the resulting potential energy well is mostly determined by the domain wall profile. A domain wall passing slowly through the SrTiO_3_ layer would experience a potential well with a reduced energy depth, because the domain wall profile would adapt to the material inhomogeneity in order to reduce the energy costs. However, we expect that such a relaxed potential energy profile would be qualitatively similar to that of Figure 7. In fact, the total potential energy density increase achieved by displacing the Bloch wall away from the pinned position at the SrTiO_3_ layer, obtained by the energy difference between the fully relaxed configurations shown in Figure 5, yields a quite considerable potential energy depth of about 6 mJ/m^2^. In our simulations, the Bloch wall placed 1–7 nm away from the defect layer always spontaneously moved back to it.

These simulations suggest that an approximately 1 nm thin SrTiO_3_ layer incorporated in ferroelectric BaTiO_3_ crystal is capable of considerably influencing the structure and properties of the 180-degree Bloch domain walls parallel to it. Since the domain wall has lower energy when located right at the SrTiO_3_ layer, one can speculate that a pair of such layers can be used as a nucleation center for favoring antiparallel ferroelectric domain with desirable crystallographic orientation of adjacent domain walls. For example, one can choose the 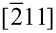 crystallographic direction, favorable for Bloch wall formation. It can also be used to pin domain walls already present in the material. Most interestingly, we have seen that the inner polarization of the Bloch wall would be substantially reduced while passing through the thin paraelectric layer. Thus, the layer acts as a bottleneck for the helicity order parameter of the wall.

## Conclusion

**Figure 8:**
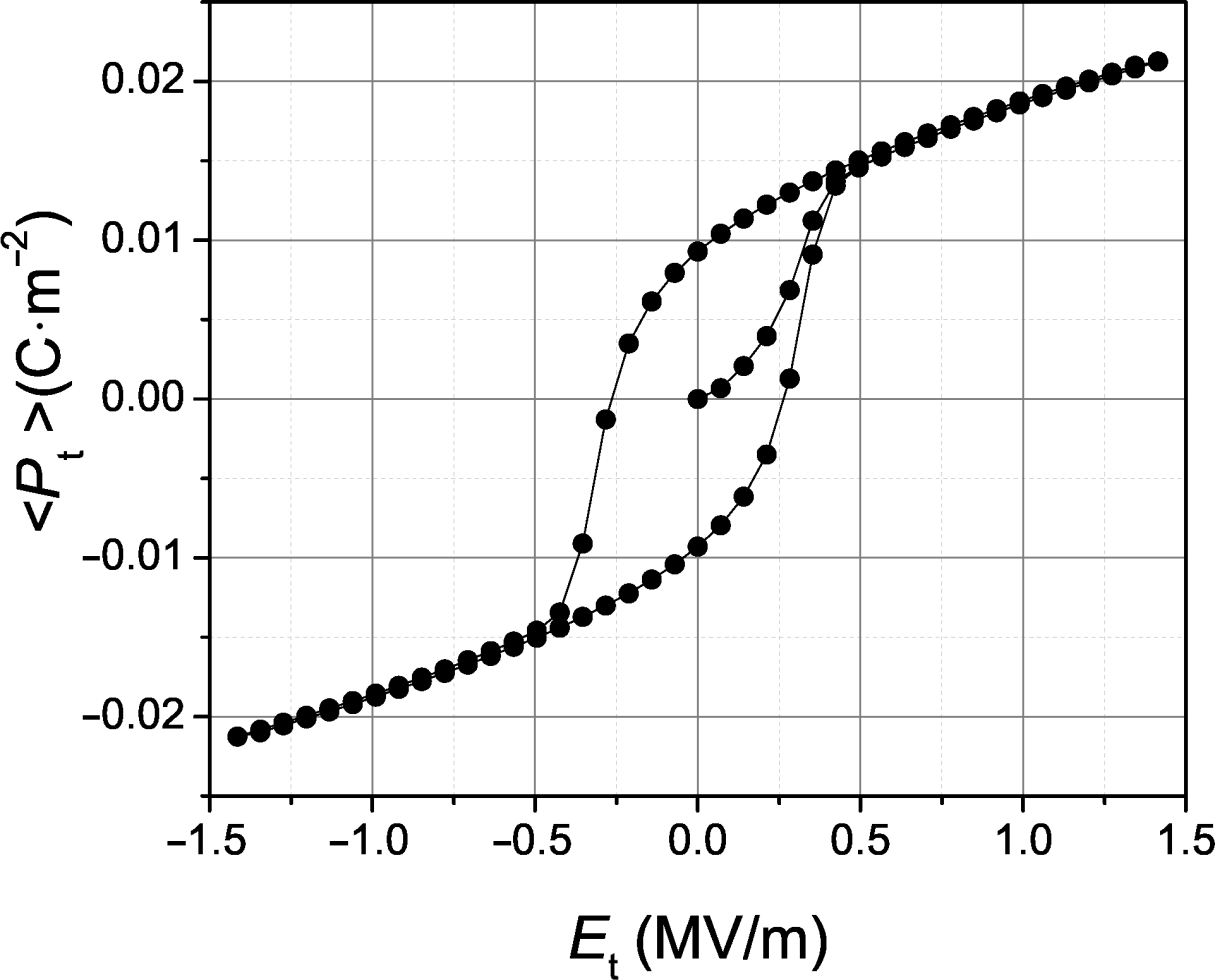
Calculated hysteresis loop demonstrating switching of the *P*_t_ component of the Bloch wall located at a 1 nm thin SrTiO_3_ layer. The indicated value of the polarization *P*_t_ is averaged over the whole supercell shown in Figure 1b.

In summary, since the Bloch character is strongly suppressed when the domain wall is right at the SrTiO_3_ layer, the layer can facilitate selection of the sign of the *P*_t_ component, and therefore, selection of the sign of its helicity. As a matter of fact, in our simulations made without a SrTiO_3_ layer, the application of an *E*_t_ electric bias to the Bloch domain wall either preserved its helicity or destroyed the wall completely. On the other hand, the Bloch domain wall located at the 1 nm thin SrTiO_3_ layer could be easily switched with a 0.5 kV/mm electric field, as is apparent from the quasistatic hysteresis loop shown in Figure 8 (see below). In fact, the thickness of the SrTiO_3_ layer can be tuned in a way that the wall passing through there is effectively in the state just below the phase transition from the Bloch to the Ising state. Then, as the domain wall passes through such a paraelectric layer, it should easily acquire the *P*_t_ component favored by even quite a moderate *E*_t_ electric bias. Thus, ferroelectric Bloch domain walls are shown to be ideal nanoscale objects with switchable properties. These findings are expected to inspire the design of functional properties of ferroelectric nanostructures.

## Calculation Details

The domain wall structures displayed in Figures 3–5 fit in a 64 × 128 × 128 equidistant point mesh with 0.5 nm lattice spacing, forming a periodic simulation box with its the edges along the principal pseudocubic crystallograpic axes (see Figure 8).

The BaTiO_3_ and SrTiO_3_ Ginzburg–Landau–Devonshire model potential parameters used in the present calculations are those of [12], except for the temperature parameter, which was set to 118 K here (low temperature is needed to drive BaTiO_3_ into the rhombohedral ferroelectric phase). Phase-field simulations for pure BaTiO_3_ single crystal and for the BaTiO_3_–SrTiO_3_ crystalline superlattice at stress-free mechanical conditions were performed using the phase-field simulation code ferrodo [8]. The approximate profile of the ferroelectric Bloch wall is known from the previous calculation and this knowledge could be conveniently used for setting the initial conditions for the present phase field simulations. In particular, the initial conditions were set in a way to favor the orientation, distance and the location of the walls in the simulation box, but there were no real constrains introduced there, only the natural limitations resulting from the standard protocol of simulated annealing procedure, governed by the time-dependent Ginzburg–Landau equation. The hysteresis loop shown in Figure 8 has been calculated quasistatically, from a sequence of configurations relaxed under fixed bias electric fields, similarly as in [12]. The initial state had two domain walls with opposite *P*_t_ values, as in Figure 3, then the electric field was gradually increased, decreased and increased again to form a whole polarization cycle. In the saturated states, *P*_t_ values in the two domain states had the same magnitude and sign.
